# Ultra-widefield fundus imaging in gas-filled eyes after vitrectomy

**DOI:** 10.1186/s12886-017-0510-7

**Published:** 2017-07-03

**Authors:** Makoto Inoue, Takashi Koto, Kazunari Hirota, Akito Hirakata

**Affiliations:** Kyorin Eye Center, Kyorin University School of Medicine, 6-20-2 Shinkawa, Mitaka, Tokyo, 181-8611 Japan

**Keywords:** Gas tamponade, Retinal detachment, Ultra-widefield imaging, Vitrectomy

## Abstract

**Background:**

To evaluate the quality of the images obtained by an ultra-widefield device in gas-filled eyes after vitrectomy for a retinal detachment.

**Methods:**

Retrospective case series. The ultra-widefield scanning laser ophthalmoscopic images (Optos 200Tx imaging system) of 40 eyes that were gas-filled with 40 to 90% of the vitreous cavity after vitrectomy for a rhegmatogenous retinal detachment were studied. The rates of detecting the rates of reattachments and the causative retinal tears that were treated and were in the superior or inferior areas in eyes with intravitreal gas of ≥60% were compared to that to eyes with intravitreal gas of <60% of the vitreous cavity. The widefield images recorded with 532 nm (green) or 633 nm (red) wavelength laser lights were compared to determine which wavelength had clearer images in 20 eyes of retinal detachment with superior retinal tears and were more than 50% gas-filled.

**Results:**

The ultra-widefield images showed a retinal reattachment in all eyes on postoperative days 1 to 40 (mean; 8.7 ± 7.5 days). A superior retinal break was not visible in 5 of 26 eyes due to a reflection from the intravitreal gas bubbles when the gas was <60%. However, the superior retinal breaks were visible when the patients were requested to gaze downward to reduce the reflection of the gas bubble. The retinal breaks treated with laser burns and the retinal vasculature were imaged better with green laser than red laser light, and the choroidal vasculature was seen better with red laser light.

**Conclusions:**

Ultra-widefield fundus images can be used to evaluate and document the retinal breaks and retinal reattachments in gas-filled eyes. The green and red laser lights can image different depths of the retina and choroid in gas-filled eyes.

## Background

A rhegmatogenous retinal detachment is a separation of the sensory retina from the retinal pigment epithelium (RPE) caused by the entry of fluid into the subretinal space through retinal tears. Rhegmatogenous retinal detachments are treated by scleral buckling, pars plana vitrectomy, or a combination of both [1–5]. Scleral buckling has been the conventional treatment for retinal detachments, but recent developments in vitrectomy have made pars plana vitrectomy a more common treatment of retinal detachments. During the vitrectomy, different types of gas are injected into the vitreous cavity to tamponade the retina, however the intravitreal gas can decrease the visibility of the retina, and reflections from the intravitreal gas bubble can make it difficult to observe and photograph the fundus.

Ultra-widefield imaging of the fundus allows clinicians to evaluate the retina far beyond the equator of the fundus in a single image [6–8]. The Optos ultra-widefield imaging system is a scanning laser ophthalmoscope which can obtain widefield images with scanning laser wavelengths of 532 nm (green) or 633 nm (red) [9]. The two images can be viewed separately or superimposed to yield a semi-realistic color image. The design of the ellipsoid mirror of the Optos makes it possible to obtain ultra-widefield images of 200 degrees horizontally without pupillary dilatation. In addition, the use of two laser wavelengths is advantageous over the conventional imaging systems because the red laser penetrates deeper into the retina and the choroid, and the green laser light provides better images of the superficial layers of the retina and retinal vessels.

Anderson and associates [7] presented a case report of a retinal detachment evaluated with the Optos ultra-widefield images prior to the surgery, after scleral buckling surgery with intravitreal gas injection, and after vitrectomy with a gas tamponade. They reported that the ultra-widefield fundus imaging system was able of delineating the extent of the retinal detachment even in the presence of intraocular gas after the vitreoretinal surgery.

The aim of this study was to evaluate the efficacy of ultra-widefield imaging of the fundus to delineate the causative and treated retinal tears and reattachments in gas-filled eyes after vitrectomy for a rhegmatogenous retinal detachment. In addition, the efficacies of green and red laser wavelengths in gas-filled eyes with superior retinal breaks were determined.

## Methods

The medical records of 40 eyes of 40 patients who had undergone vitrectomy with a gas tamponade for a retinal detachment were reviewed. The patients were examined by indirect ophthalmoscopy and slit-lamp biomicroscopy with a non-contact or contact wide angle lens to detect all retinal breaks preoperatively, and all the retinal breaks were treated with endolaser during the vitreous surgery. Ultra-widefield scanning laser ophthamloscopic images (Optos 200Tx imaging system, Optos PLC, Dunfemline, Scotland, UK) were recorded from dilated eyes in the primary position after the vitrectomy. The eyes were filled with different types of gas with a volume of 40% to 90% of the vitreous cavity.

In the initial investigation, the rate of detecting the causative and treated retinal tears and reattachments with intravitreal gas to ≥60% of the vitreous cavity was compared to that with gas of <60% of the vitreous cavity. The ability to detect the retinal reattachments was evaluated in eyes with retinal breaks in all quadrants but the detection of the retinal breaks was evaluated in eyes with retinal breaks only in the superior or inferior areas. A positive identification of retinal breaks was defined as the identification of all the retinal breaks in a quadrant in eyes with multiple retinal breaks. The identification was made by two retinal specialists (MI, TK) who were masked to the patients’ information including the locations and numbers of the retinal breaks. The rate of detecting retinal tears and retinal reattachment in the images were evaluated. When the decision was not the same, a third investigator (KH) examined and discussed the findings to make the final decision.

For the second investigation, the efficacy of the red and green wavelengths in evaluating the retina in gas-filled eyes was compared. To avoid the effects of the location of the retinal breaks and the volume of the gas, the ultra-widefield images of 20 eyes filled with a gas volume of more than 50% of the vitreous cavity and with superior retinal breaks were rewiewed. These images were divided into two identical images taken with the 532 nm or 633 nm laser lights and exported as black-and-white images. The two images were examined to determine which wavelength gave better images of the superior retinal breaks and the retinal and choroidal vasculatures. The ability to detect superior retinal breaks and retinal and choroidal vasculatures through the intravitreal gas was scored into 3 grades; 2 = clearly detected, 1 = moderately detected, 0 = barely or not detected. The scoring was made by two investigators (MI, TK) who were masked to the patients’ information including the location and number of the retinal breaks.

Vitreous surgery was performed with 25-gauge instruments, and the retina was tamponaded after the vitrectomy with air, 20% sulfur hexafluoride (SF_6_), or 14% perfluoropropane (C_3_F_8_) gas. The volume of intravitreal gas was determined by one of the authors (MI) based on the level of the inferior gas meniscus at the retina observed with an indirect ophthalmoscope in a sitting position [10, 11].

## Results

**Fig. 1:**
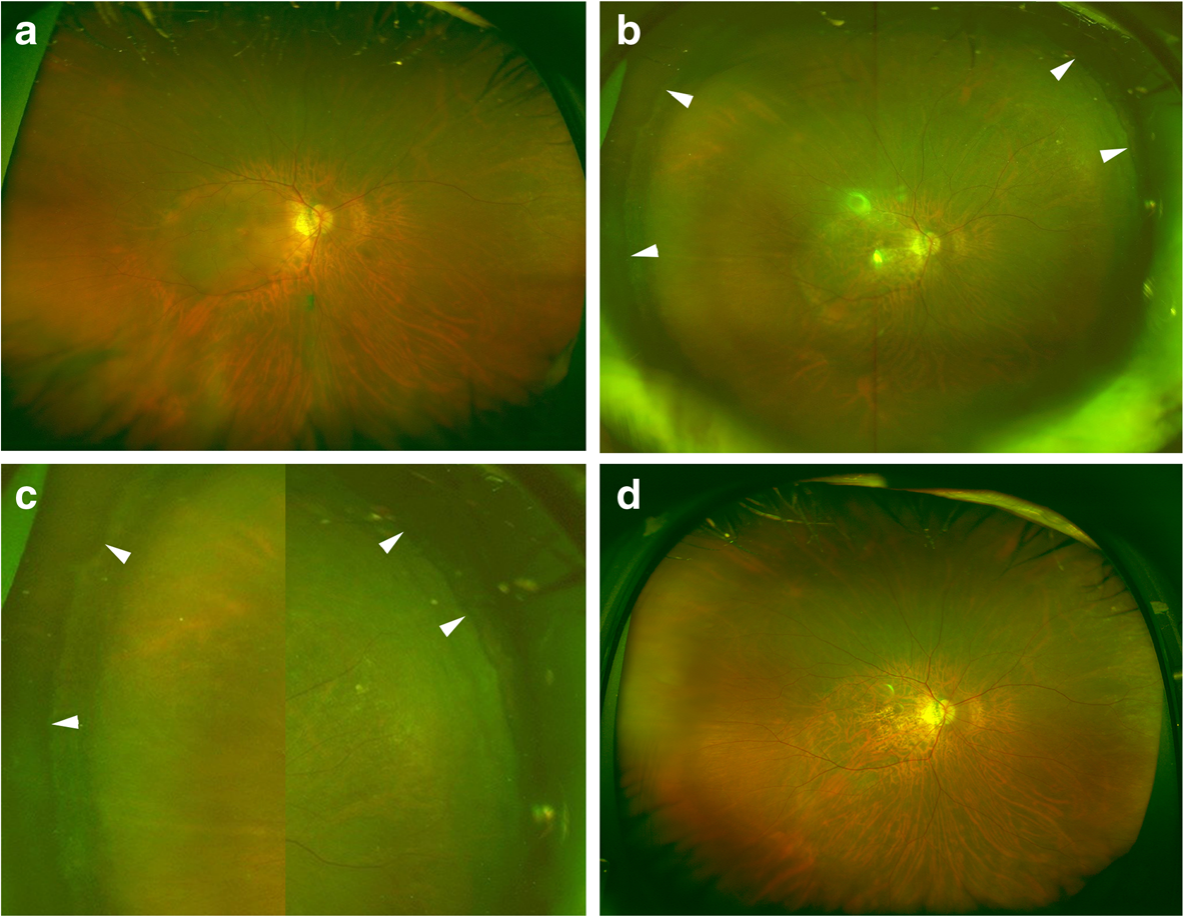
Preoperative and postoperative ultra-widefield fundus images of an eye with a macular hole retinal detachment. **a** Preoperative ultra-widefield image showing macular hole retinal detachment within the vascular arcade. **b**: Postoperative image taken on the day after vitrectomy with 80% intravitreal gas showing that the retina is reattached. The ora serrata (white arrowheads) and ciliary epithelium can be seen through the gas. **c** Magnified images of Fig. **b** showing that the ora serrata (white arrowheads) and ciliary epithelium are visible. **d** Ultra-widefield image taken 6 months postoperatively shows retinal reattachment without gas tamponade, but the ora serrata and ciliary epithelium are not visible

Combined cataract surgery was performed on 29 eyes, lens-sparing vitrectomy was performed on 3 eyes, and 8 eyes were pseudophakic. Scleral shortening for macular hole retinal detachment was performed on one eye [12], and laser in-situ keratomileusis (LASIK) had been performed on one eye prior to the vitrectomy. The Optos ultra-widefield images showed that the retina was reattached in all eyes regardless of the volume of the gas in the vitreous cavity. The Optos ultra-widefield images showed that the retina was reattached even in the 15 eyes filled with gas volumes of 80 to 90% (Fig. 1). Clear images were also obtained from one eye with severe nystagmus due to the morning glory syndrome and from one eye of a patient with intellectual disability. No new retinal breaks were found ophthalmoscopically or in the ultra-widefield images of the gas-filled eyes postoperatively. The detections of retinal breaks and retinal reattachments were identical for the two investigators (interclass correlation coefficient (2,1), *r* = 1.00).

### Identification of sites of the causative retinal tears and retinal reattachments

Ultra-widefield images were taken on postoperative days 1 to 40 with a mean ± standard deviation of 8.7 ± 7.5 days. The retinal tears were located in the superior retina in 20 eyes, superior and inferior sectors in 6 eyes, the superior-temporal quadrant in one eye, temporal sector in one eye, inferior sector in 3 eyes, peripapillary area in one eye, and in the macular area in 8 highly myopic eyes with a macular hole retinal detachment. The rate of detecting tears in the superior area was significantly higher in eyes with a gas volume of ≥60% (100%; 11 of 11 eyes) when the eyes were in the primary position than in eyes with gas volume < 60% (67%; 10 of 15 eyes, *P* = 0.046, Fisher’s exact probability test, Table 1). In the primary position, the incidence of detecting retinal tears in the inferior area of eyes with gas volume of ≥60% (71%; 5 of 7 eyes) was not significantly different from that in eyes with gas volume of <60% (100%; 2 of 2 eyes, *P* = 0.583, Fisher’s exact probability test). Retinal breaks in the superior area were not detected in 5 of 26 eyes due to reflections from the intravitreal gas bubble, and all of these eyes had intravitreal gas volume of <60%. When the size of the gas bubble was smaller, the superior retinal breaks in 6 eyes were difficult to detect in the primary position due to the strong reflections of the gas bubble. However, the retinal breaks in these 6 eyes could be detected when the patients were asked to gaze downward to reduce the reflections from the intravitreal gas bubbles (sensitivity = 100%, specificity = 100%). Inferior retinal breaks were detected in 2 eyes when the gas bubble did not cover the retinal breaks and 5 eyes through the gas bubble. In 2 other eyes, the inferior retinal breaks were not detected due to the reflection from the intravitreal gas bubble (sensitivity = 78%, specificity = 100%). The macular hole was not detected in all of the 8 eyes with a macular hole retinal detachment.

**Table 1:** The location of the retinal breaks and the volume of intravitreal gas

Locations of retinal breaks	gas ≥60%	gas <60%	*P* value^a^
Superior area	11/11 eyes (100%)	10/15 eyes (67%)	0.046
Inferior area	5/7 eyes (71%)	2/2 eyes (100%)	0.583

^a^Fisher’s exact probability test

**Fig. 2:**
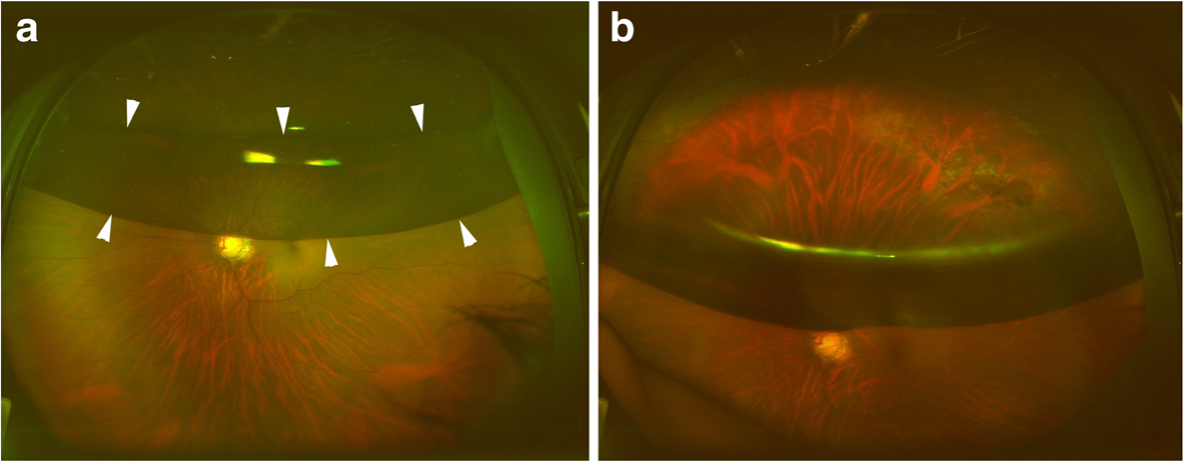
Postoperative ultra-widefield images of an eye with a superior bullous retinal detachment. **a**: Ultra-widefield image on postoperative day 6 showing a intravitreal gas bubble with superior and inferior areas. The superior oval area originates from the image of the superior retinal break seen thorough the gas bubble but the inferior banana-shaped area (white arrowheads) with a mirror image of the inferior retina originates from the reflection of the inferior retina. **b**: Superior retinal breaks with laser burns around the lattice degeneration are seen clearer in the same eye of Fig. **a**, and the intravitreal gas becomes a single-layered bubble with a view of the ora serrata (white arrowheads) when the patient looks downward

The intravitreal gas was seen as a double-layered image with superior and inferior areas in 17 of 17 eyes with intravitreal gas volumes of 40 to 50% (Fig. 2). The two areas were not seen in 11 eyes with intravitreal gas of 80 to 90% which was significantly fewer than that with gas of 40 to 50% (*P* < 0.0001, Fisher’s exact probability test). The superior oval area of the gas bubble originated from the image seen through the gas bubble but the inferior, banana-shaped area, originated from the mirror images by the reflection from the inferior retina and not through the gas bubble. The mirror images through the inferior banana-shaped area was confirmed in 15 eyes by the pattern of the retinal vasculature.

### Comparisons of images with green and red laser lights in gas-filled eyes

**Fig. 3:**
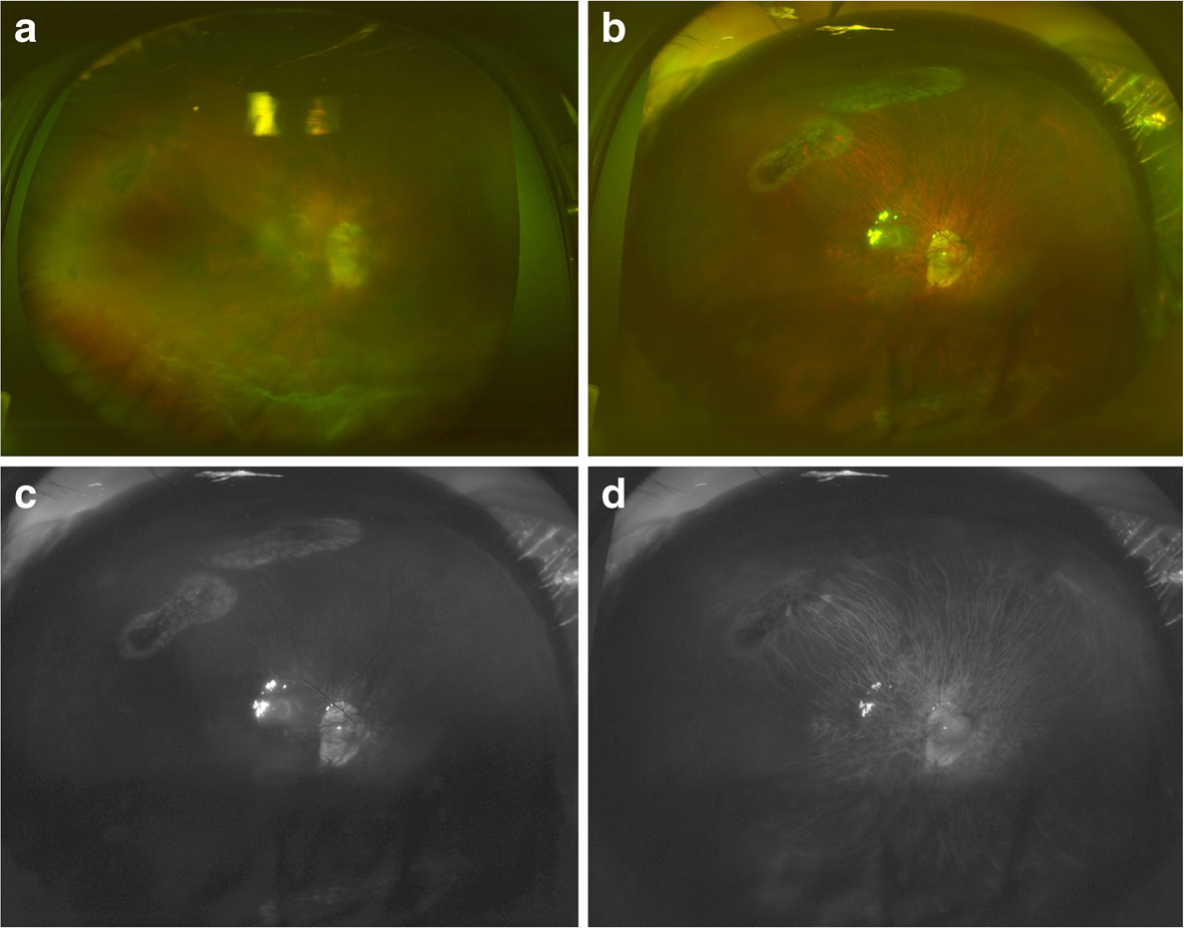
Preoperative and postoperative ultra-widefield images with total retinal detachment in a patient with intellectual disability. **a** Preoperative ultra-widefield image shows total retinal detachment. **b** Postoperative image showing that the retina is reattached. Laser burns around the lattice degeneration can be seen under the intravitreal gas. **c** Postoperative image of green laser of Fig. **b** shows retinal reattachment with laser burns, retinal vessels, and some reflection of the intravitreal gas. **d** Postoperative image of red laser of Fig. **b** shows retinal reattachment with less visible laser burns and less reflection of gas, but choroidal vessels are seen more clearly

Superior retinal breaks were identified in 18 of 20 eyes through the intravitreal gas with the green laser wavelength and in 17 eyes with red laser wavelength (Fig. 3, *P* = 0.50, Fisher’s exact probability test). In the other 2 eyes with superior retinal breaks, a retinal reattachment was identified but the retinal breaks were not detected in both the green and red laser wavelength images.

The score for identifying superior retinal breaks was 1.6 ± 0.5 (mean ± standard deviation) with the green laser which was significantly higher that with the red laser with a score of 0.5 ± 0.5 (*P* < 0.001, Mann Whitney U test). The score for retinal vasculature with the green laser was 1.4 ± 0.6 which was also significantly higher than that with the red laser at 0.7 ± 0.6 (*P* = 0.002). The score for choroidal vasculature was 0.1 ± 0.3 with the green laser which was significantly lower than that with the red laser at 1.7 ± 0.6 (*P* < 0.001).

## Discussion

The ocular fundus has been traditionally examined by monoocular or biocular indirect ophthalmoscopy in combination with detailed drawings or panoramic photographs with a fundus camera. Ultra-widefield images of the fundus have been recently recorded with the Optos system, and these images can determine the extent of a retinal detachment even in the presence of 80 to 90% intraocular gas as was found in this study. Ultra-widefiled imaging has been used to record retinal detachments, monitor their repair, and follow the retinal status postoperatively [7].

**Fig. 4:**
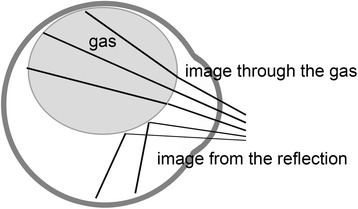
Schema of retinal images through the intravitreal gas. The images through the intravitreal gas consists of two components, the image of the retina through the gas in the superior area and the mirror image of inferior retina from the reflection of the gas surface in the inferior area

Ultra-widefield fundus imaging is based on scanning laser technology in combination with a large ellipsoidal mirror. We assumed that scaning with a shorter wavelength laser light might increase the reflection of intravitreal gas, but the fundus in the ultra-widefield images was clearly visible through the intravitreal gas with both wavelengths. Confocal scanning may reduce the reflection from the intravitreal gas. The images through the intravitreal gas consisted of two components, the image of the retina through the gas in the superior area and the mirror image of the inferior retina from the reflection of the gas surface in the inferior area (Fig. 4). The images through the intravitreal gas was minified which enabled the ultra-widefield images including a view to the ora serrata in some of the gas-filled eye (Fig. 1b) but not through the vitreous fluid. The intravitreal gas was seen to be oval-shaped and the inferior area as banana-shaped. The Optos ultra-wide field images have been described to be stretched by 1.12-fold in the horizontal direction with respect to the vertical direction [13]. The ellipsoid mirror of the Optos ultra-widefield system may produce these horizontally elongated images that are oval- and banana-shaped. The banana-shaped mirror images were seen when the intravitreal gas became less than 80% of the vitreous cavity and a larger intravitreal gas bubble hid this mirror image below the image through the gas. We should be aware of this type of artifact in evaluating gas-filled eyes.

The red wavelength laser scans had better penetration into the deeper layers of the retina and the choroid, and green wavelength gave better images of the surface layers of the retina and laser burns [9]. The diagnostic capabilities of ultra-widefield imaging with two wavelength lasers have been suggested to be a potential method to differentiate malignant ocular tumors from nonmalignant lesions with high diagnostic accuracy in clinically-diagnosed melanocytic choroidal tumors [9]. As in gas-filled eyes, two laser scans enabled an evaluation of retinal images while maintaining the beneficial features of each wavelength. However, we evaluated only eyes with superior retinal breaks because the detection of inferior retinal breaks was more dependent to the volume of the intravitreal gas. A macular hole was not detected in all highly myopic eyes with a macular hole retinal detachment. This may be becase we did not perform laser photocoagulation on the macular hole, and the macular hole was often surrounded by depigmented chorioretinal atrophy.

The ultra-widefield system can also obtain fluorescein angiograms and has been used to evaluate eyes with diabetic retinopathy, uveitis, retinal vascular occlusion, and retinopathy of prematurility [14–23]. Ultra-widefield fundus autofluorescence imaging has also been used to evaluate peripheral retinal detachments, age-related macular degeneration, and retinitis pigmentosa [24–27]. We have not used these methods in gas-filled eyes but they may be feasible because the effects of intravitreal gas was minimal in the ultra-widefield images.

The extremly large depth of focus of the ultra-widefield system allows the peripheral retina, intraocular gas bubble, and posterior pole to be in focus simultaneously in gas-filled eyes. A faster scan with green and red lasers and an ultra-widefield images with a greater depth of focus may allow the detection of retinal images in eyes with nystagmus or in patients with intellectual disability. However, it may be affected by peripheral lens opacities, corneal opacities including intracorneal implantation of corneal inlay, cilia, and eyelids [28]. Postoperative cataracts caused by intravitreal gas in phakic eyes can reduce the clarity of the ultra-widefield images temporarily.

There are limitations in this study. This was a retrospective case series conducted in a single academic hospital. The number of patients was small and each patient had a different type of retinal detachments and retinal breaks. The grading was essentially subjective which might have affected the results. Therefore, prospective studies of randomized eyes with larger sample sizes may be needed to confirm these results.

## Conclusions

The ultra-widefield fundus imaging is useful in evaluating postoperative retinal breaks and retinal reattachments in gas-filled eyes. The two wavelength light sources maintain the efficacy of different scanning depth can also be used in gas-filled eyes. These advantages are useful in documenting the clinical course before and after vitreous surgery with gas tamponade.

## Abbreviations

C_3_F_8_: Perfluoropropane
LASIK: laser in-situ keratomileusis
RPE: Retinal pigment epithelium
SF_6_: Sulfur hexafluoride
